# Scheduled Daily Mating Induces Circadian Anticipatory Activity Rhythms in the Male Rat

**DOI:** 10.1371/journal.pone.0040895

**Published:** 2012-07-25

**Authors:** Glenn J. Landry, Hanna Opiol, Elliott G. Marchant, Ilya Pavlovski, Rhiannon J. Mear, Dwayne K. Hamson, Ralph E. Mistlberger

**Affiliations:** Department of Psychology, Simon Fraser University, Burnaby, British Columbia, Canada; University of Pennsylvania School of Medicine, United States of America

## Abstract

Daily schedules of limited access to food, palatable high calorie snacks, water and salt can induce circadian rhythms of anticipatory locomotor activity in rats and mice. All of these stimuli are rewarding, but whether anticipation can be induced by neural correlates of reward independent of metabolic perturbations associated with manipulations of food and hydration is unclear. Three experiments were conducted to determine whether mating, a non-ingestive behavior that is potently rewarding, can induce circadian anticipatory activity rhythms in male rats provided scheduled daily access to steroid-primed estrous female rats. In Experiment 1, rats anticipated access to estrous females in the mid-light period, but also exhibited post-coital eating and running. In Experiment 2, post-coital eating and running were prevented and only a minority of rats exhibited anticipation. Rats allowed to see and smell estrous females showed no anticipation. In both experiments, all rats exhibited sustained behavioral arousal and multiple mounts and intromissions during every session, but ejaculated only every 2–3 days. In Experiment 3, the rats were given more time with individual females, late at night for 28 days, and then in the midday for 28 days. Ejaculation rates increased and anticipation was robust to night sessions and significant although weaker to day sessions. The anticipation rhythm persisted during 3 days of constant dark without mating. During anticipation of nocturnal mating, the rats exhibited a significant preference for a tube to the mating cage over a tube to a locked cage with mating cage litter. This apparent place preference was absent during anticipation of midday mating, which may reflect a daily rhythm of sexual reward. The results establish mating as a reward stimulus capable of inducing circadian rhythms of anticipatory behavior in the male rat, and reveal a critical role for ejaculation, a modulatory role for time of day, and a potential confound role for uncontrolled food intake.

## Introduction

Daily feeding schedules that limit caloric intake to a specific time of day induce food anticipatory activity rhythms in rats, mice and a variety of other species. Food anticipatory rhythms exhibit formal properties of circadian clock control, including persistence for several cycles during total food deprivation, gradual resetting following shifts of mealtime, and a limited range of entrainment [1]–[4]. These properties are distinct from those of anticipatory behaviors observed under fixed interval feeding schedules in the seconds to minutes range, and are thus assumed to be mediated by different mechanisms [5]. Food anticipatory rhythms can be generated at any time of the LD cycle, and are not affected by ablation of the master light-entrainable circadian pacemaker in the suprachiasmatic nucleus (SCN) [6], [7]. Many other brain regions and peripheral organs and tissues exhibit a capacity for intrinsic circadian rhythmicity, and most of these local rhythms can be shifted and entrained by daily feeding schedules [8]–[10]. However, the location of circadian oscillators hypothesized to drive food anticipatory activity rhythms is uncertain [11], [12].

Anticipatory rhythms have also been observed in rats, hamsters or mice in response to daily schedules of restricted access to water [13], salt [14], palatable, calorically dense snacks [15]–[18] and psychomotor stimulant drugs of abuse [19]–[21]. These observations suggest that anticipatory rhythms may be generated by any stimulus that activates neural reward circuits, and that circadian oscillators driving anticipatory rhythms may be located in neural circuits mediating reward or may be entrained by neural correlates of reward [18], [22]–[24]. Consistent with this hypothesis, circadian clock genes are expressed in midbrain and forebrain components of the reward system, and expression can be induced or shifted by natural rewards and dopaminergic compounds [8], [9], [18], [25]. Evidence against this hypothesis is that rats and mice often fail to anticipate palatable foods [26]–[28], even when the amount of food ingested is sufficient to support classical and operant conditioning and to activate mesocorticolimbic reward circuits [29]. Other known reward stimuli that failed to induce anticipatory activity rhythms in one or more studies include palatable but non-caloric ‘foods’ [15], daily timed wheel access [30]–[32], and daily injections of morphine [33]. These results suggest an alternate hypothesis, that the circadian mechanism timing anticipatory rhythms may be instantiated in neural circuits regulating water, salt or energy homeostasis, and may not be entrained by reward system activation. Rhythms that can be induced by psychomotor stimulants may also be dependent on the metabolic consequences rather than the reward effects of those compounds [34], [35].

To further evaluate reward as a stimulus for inducing circadian anticipatory rhythms, we asked whether male rats can anticipate a daily opportunity to mate with female rats in behavioral estrous. Copulation is a powerful natural reward, by some measures the most powerful, and unlike food, water, and salt ingestion, is not homeostatically regulated. It is thus conceivable that scheduled daily copulation could induce circadian anticipatory rhythms that are more robust and that persist for more cycles in constant conditions than do anticipatory rhythms induced by other reward stimuli. A paper published while this study was in progress provided evidence that male mice can anticipate a daily opportunity to mate [36]. By contrast with anticipation of food, anticipation of mating in that study was relatively weak and not apparent in all mice, possibly because the hormonal state of the female mice was not controlled or because the mating time corresponded to a nadir in a daily rhythm of sexual reward [37]. Our results confirm and extend these findings, by demonstrating anticipation of a daily mating opportunity in male rats, and by establishing that ejaculations are critical, that time of day plays an important modulatory role, and that post-copulatory food intake can play a confound role. We also observed a position bias during nighttime anticipation but not daytime anticipation, which may reflect a daily rhythm in sexual reward, previously defined on the basis of conditioned place preferences [37].

## Materials and Methods

### Animals and Apparatus

All animal work was conducted according to guidelines established by the Canadian Council on Animal Care and was approved by the University Animal Care Committee at Simon Fraser University (permit number 1004). Adult male and female Sprague-Dawley rats (Charles River, PQ) were housed in climate controlled vivarium rooms with fluorescent lighting (LD 12∶12, ∼200 lux). In Experiments 1 and 2, the male rats were housed individually in cages with three compartments. The ‘home’ compartment was a standard polypropylene cage (45×24×20 cm) with a wire floor over a metal waste tray, and a standard top with metal bars, a hopper for rodent chow pellets (formulation #5001), and a bottle holder. This home compartment was connected by PVC tubes to a Wahmann running wheel (35 cm diameter} on one side, and to a solid bottom polypropylene ‘mating’ cage (45×24×20 cm} with corn cob bedding on the opposite side. Running wheels are commonly used in studies of circadian food anticipatory activity, because anticipatory rhythms in running are more precise and of higher amplitude than are other activity measures. We wished to exploit this advantage, and as much as possible follow procedures used in studies of scheduled feeding. The running wheel access tube (10×20 cm} was large enough for use as a sleeping tube or for light avoidance, while the mating cage access tube (7.5×12 cm} was smaller, discouraging its use as a sleeping tube. In Experiment 3, the running wheel and its connecting tube were replaced by duplicates of the mating cage and its connecting tube. The duplicate cage contained used litter refreshed daily from the mating cage, which could be seen and smelled from within the access tube, but was not otherwise accessible. Access to the mating cage was controlled by a manually operated vented sheet metal gate, which was opened only at the beginning and ending of daily mating sessions. The cage complexes were housed 1 or 2 per shelf in open cabinets. In Experiments 1 and 2, a group of male rats were housed individually in standard polypropylene cages with bedding and overhead motion sensors, in a separate cabinet in the same room. These rats were not exposed directly to female rats. Females rats were individually housed in standard polypropylene cages in a separate room.

In Experiment 1, general activity was detected using a passive infrared motion sensor (Quorum RR-150} positioned 25 cm above the center of the home cage. Wheel revolutions were detected via microswitch closures. In Experiment 2, a third motion sensor was installed inside the access tube to the mating cage, to detect activity specifically directed at the mating cage gate. In Experiment 3, the running wheel was removed, and a motion sensor was installed in the tube connecting to the locked cage containing mating litter. All sensors and switches were monitored continuously using the ClockLab data acquisition system (Actimetrics, IL., USA}. Activity counts were summed at 1-minute intervals. Locomotor activity of female rats was not recorded.

### Surgery and Steroid Replacement

Female rats were anesthetized with halothane for ovariectomy and implantation of Silastic tubing capsules (1.57 mm inner diameter×3.18 mm outer diameter; Dow Corning} containing b-estradiol 3-benzoate (2–3 mm in length; Sigma E8515}. The capsules were implanted in a small pocket created under the dermis, between the neck and shoulders.

### Experiment 1. Scheduled Daytime Mating with Food and Running Wheel Available *ad-libitum*

**Figure 1:**
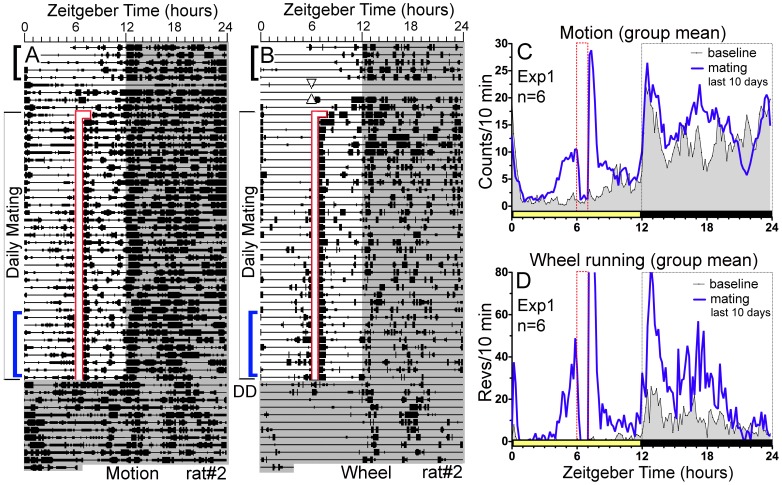
Experiment 1: Rats anticipate scheduled daytime mating with food and a running wheel continuously available. Panels A–B. Actograms of home cage motion (A} and wheel running (B} of a representative rat. Each line represents one day of recording, plotted in 10 minute bins from left to right, with consecutive days aligned vertically. Bins in which activity counts were registered are represented by heavy bars (in quartile weights}. Daily scheduled mating time is indicated by the vertical opaque bar outlined in red. Lights-off is indicated by grey shading. The triangles in panel B denote the 48 h during which the rats were confined to their mating cage to gain sexual experience. The home cage motion sensors could detect activity in some parts of the mating cage. Panels C–D. Group mean waveforms of home cage motion (C} and wheel running (D}, averaged over 5 baseline days (shaded curve, from days indicated by the upper bracket to the left of panels A and B} and 10 scheduled mating days (heavy blue curve, from days indicated by the lower bracket to left of panels A and B}. Zeitgeber time refers to the hour of the LD cycle (lights-off from ZT12-24}. Mate access time (ZT6-7} is denoted by the vertical bar (red dotted bars}.

Male rats (N = 6, ∼ 3 months age} were housed in the multi-compartment cages with free access to food, water and running wheels for 8 days. The sequence of experimental conditions is illustrated graphically in Figures 1a,b. To gain mating experience prior to scheduled mating, on days 6 and 7 the rats were confined to their mating cages and paired with steroid-primed estrous female rats. After 2 recovery days, a daily mating schedule was initiated. For 36 days, in the middle of the light period, at Zeitgeber Time (ZT} 6 (where ZT0 is defined as lights-on}, a steroid-primed estrous female rat was placed in the mating cage, the access gate was lifted and the male rat ushered through the access tube into the mating cage. To induce estrous, the female rats (N = 12} received progesterone (500 mg in 0.1 cc corn oil s.c.; Sigma P0130} every 4^th^ day, 4 h prior to mating time (individual females were used only on the days that they received progesterone priming}. Return access to the home cage was blocked during the mating hour. After 10 minutes, the female was removed and the male was allowed to rest or explore the mating cage. Bedding was not changed. A second female was then introduced for 10 minutes, followed by a second 10 minute rest break, and introduction of a third female for a final 10 minutes. Thus, each male could mate with up to 3 females during the same hour each day. Females could mate with up to 3 males in a session, and where then rested for 3 days. Mating activity was monitored continuously by two trained observers scanning 3 cages each. Intromissions and ejaculations were counted in real time for each male/female pairing according to standard criteria [38]. Female behavior was not scored in this experiment, but lordosis and proceptive behaviors such as hopping, darting and ear wiggling were observed daily [39]. During the last week of testing, chow pellets were weighed at ZT6 and ZT12 for 2 days, to determine how much the male rats ate following the mating hour. After the last day of scheduled mating, the LD cycle was replaced by constant very dim red light (DD} for a final 12 days of recording, with brief disturbances every few days for cage servicing.

### Experiment 2. Scheduled Daytime Mating with Post-coital Feeding and Running Prevented

**Figure 2:**
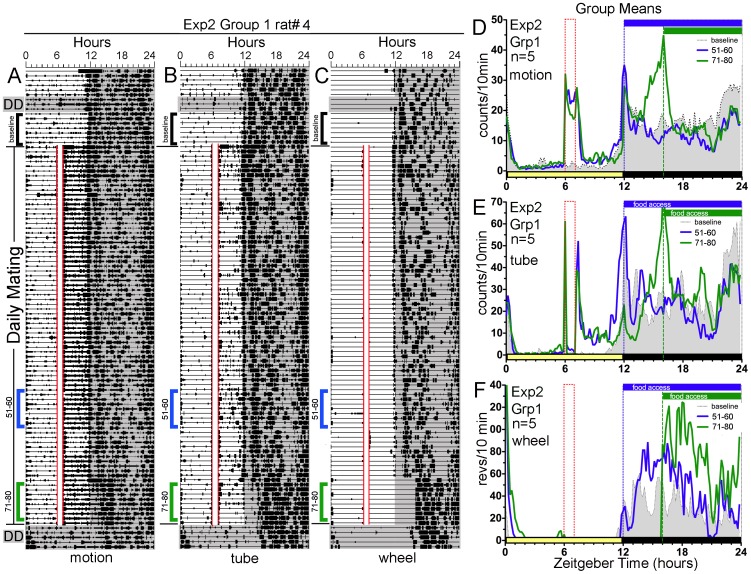
Experiment 2: Most rats fail to anticipate daytime mating when post-coital eating and wheel running are prevented for 6 or 10 h. Panels A–C. Actograms of home cage motion (A}, mating tube entry (B} and wheel running (C} of a representative rat. Panels D–F. Group mean waveforms of the 5 of 6 rats in Group 1 that showed little (N = 1} or no (N = 4} sex anticipatory activity in home cage motion (D}, mating tube entry (E} and wheel running (F}. The filled (grey} curve represents baseline prior to scheduled mating (the average of the days marked by the upper black bracket to the left of each actogram in Panels A–C}. The heavy (blue} curve represents scheduled mating days 51–60 (the middle blue brackets in A–C, when running wheels were locked from ZT6-12, and food was available from ZT12-24}. The green curve represents days 71–80 (the lower green brackets in A–C, when running wheels were locked from ZT6-16, and food was available from ZT16-24}. The time of food availability is indicated by the horizontal blue (ZT12-24} and green (ZT16-24} bars at the top of panels D–F. See Figure 1 for other plotting conventions.

Male rats (N = 18} were randomly assigned to 1 of 3 groups of 6. Group 1 and 2 rats were housed in the multi-compartment cages, while Group 3 rats were housed in single polypropylene cages. The rats were first provided access to a steroid-primed female in the mating compartment of their cage complex, every second day for 10 days. The sequence of experimental conditions after this is illustrated graphically in Figure 2a-c. During the first 5 days of activity recording, food was available only during the 12 h dark period (ZT12-24}. Food was then available ad libitum for 3 days in constant dark. This was followed by 7 days in LD with food available again only at night. For the next 80 days, Group 1 rats were provided access to 3 steroid-primed estrous females in succession each day from ZT6-7, with 10 minute rest periods as in Experiment 1. Intromissions and ejaculations were again counted in real time by trained observers. Food was available only at night. Group 2 rats were subjected to the same procedure but were prevented from entering the mating cage. During the mating hour, the females rats were transferred between the Group 1 and Group 2 mating cages every 10 minutes, so that while the Group 1 rats rested for 10 minutes, the Group 2 rats were exposed to the sight, sound and smell of an estrous female at close range. When the females were in the Group 1 mating cages, the Group 2 male rats were allowed into their mating cage for direct contact with bedding perfumed by the estrous females. Throughout this phase of the experiment, after the mating hour, Group 1 and 2 rats did not have access to either food or the running wheels from ZT7-12. During the last 10 days of the mating schedule, food and wheel access were delayed by a further 4 h, so that food was available only from ZT16-24 (8 h daily}, and running wheels from ZT16-ZT6 (14 h daily}. After the 80^th^ day of scheduled mating, the rates were recording for 5 days in constant dark with *ad-libitum* access to food and running wheels and no exposure to females. Group 3 rats were maintained on the same feeding and lighting schedules as Groups 1 and 2, but were exposed only to room sounds and smells, and had no close or visual contact with females.

Female rats (N = 46} were steroid-primed every 5^th^ day, following the protocol described for Experiment 1. Additional females were kept ready and rotated into service as needed, to ensure that all females were receptive during the mating hour. All males and females used in both experiments were confirmed to engage in mating behavior prior to use for scheduled mating.

### Experiment 3. Daytime vs Nighttime Mating: Anticipation and Place Preference

Male rats (N = 8} were housed in multi-cage complexes consisting of a home cage, two mating-style cages with connecting tubes, and no running wheel (2 of 8 cages were equipped with an overhead motion sensor, but no sensors in the tubes}. On Days 1–10, food and water were available *ad-libitum*. Access to steroid-primed females in the mating cage was provided during the first 3 nights to confirm that all males could mate to ejaculation. For the next 28 days, each rat was provided access to a single steroid-primed female for up to 60 minutes in the dark period, starting at ZT21 (9 h after lights-off}. If intromission did not occur within the first 15 minutes, or ejaculation within the first 25 minutes, the female rat was replaced by another female rat that had already successful mated that day. When ejaculation occurred the female was removed and the male left for up to 15 minutes in the mating cage, to promote place conditioning. Food was removed at ZT21 for 3 h, to prevent eating immediately following the mating sessions. After day 28, the rats were recorded for two days without mating. Mating sessions then resumed at ZT6, in the light period, for another 28 days. On day 15, the rats were left undisturbed in LD. After day 28, the rats were left undisturbed for 3 days in DD. Mating sessions for 6 of 8 rats were videotaped for analyses offline, to quantify intromission and ejaculation latencies and numbers. Female rats (N = 18} were steroid-primed every other day, following the protocol described for Experiment 1. Receptivity of 6 randomly selected female rats was scored from videotapes of two ZT6 daytime sessions and two ZT21 nighttime sessions each. The lordosis quotient was the ratio of the number of lordoses observed to the number of mount attempts [40}. Lordosis intensity was scored on a 4 point scale based on position of the head and dorsiflexion of the spine when clearly visible (0 =  arched back, head pointing down, 1 =  flat back/head, tail slightly raised, 2 =  curved back, i.e., dorsiflexion of the spine, head elevated, 3 =  dorsiflexion, head pointing straight up}.

### Data Analysis

Activity data were summed into 10 minutes bins separately for each variable and plotted as ‘actograms’ and average waveforms, using Circadia (Behavioral Cybernetics} and Prism 5.0 (Graphpad Software Inc., USA} software. Activity during the 3 h immediately prior to the mating hour (ZT3-6 or ZT18-21} was summed and expressed as total counts and ‘anticipation’ ratios (percent of total daily activity, excluding the 6 h or 3 h between mating time and lights-off or lights-on, respectively}. Group differences were evaluated using ANOVA and t-tests or Mann-Whitney U tests. Mean values in the text are reported ± SD, while means in the graphs are plotted ± SEM.

## Results

### Experiment 1

Experiment 1 assessed whether daytime mating can induce anticipatory behavior in male rats, with no restriction on post-copulatory running or eating. During the scheduled daily mating hour, all 6 male rats showed multiple mounts and intromissions and occasional ejaculations. The number of intromissions observed increased from <1/rat on day 1 to an average of 37±14/rat/session after day 2 (mean daily range 22±11 to 56±18 across rats}. These were distributed across the 3 successive female partners on most days. The number of ejaculations observed averaged 0.33±.25/rat/session.

Prior to the start of scheduled mating, all rats were strongly nocturnal in both home cage activity (82±2% occurring from ZT12-24; Fig. 1a,c} and wheel running (90±1%; Fig. 1b–d}. Daytime activity from ZT3-6 accounted for only 2.1±2.6% of total daily home cage activity, and 0.2±0.3% of total daily wheel running. During the last 10 days of scheduled mating, activity from ZT3-6 increased significantly in all rats, to 8.4±4.6% of the daily total for motion counts and 12.7±12.6% for wheel running (p<.001, by contrast with the last 5 baseline days, for each rat and both variables}. Following the last day of scheduled mating, the rats were left undisturbed in DD for a week. Anticipatory activity in the motion sensor measure persisted for about 4 circadian cycles in all 6 cases before dissipating (e.g., Fig. 1a}. The amount of anticipatory activity (ZT3-6 total} detected by this sensor increased by 210% on the first day of DD by contrast with the average amount during the last 5 days of LD (t_(5}_ = 3.59, p = .015}. Anticipatory wheel running also persisted for several days in DD, but the amount was within 2% of that evident in LD (e.g., Fig. 1b}.

Immediately following the daily mating hour, 5 of 6 rats showed a short but intense bout of wheel running, averaging ∼160 revolutions during the first 10 minutes, which was double the peak level of activity observed in any 10 minute time bin at night (Fig. 1b,d}. Food consumption from the end of the mating hour to lights-off (ZT7-12} averaged 8.4±1.9 g, representing 27±5% of total daily food intake. This is approximately the amount of caloric intake associated with anticipation of a midday palatable high-caloric snack [15}. A group of control male rats averaged significantly less food intake from ZT7-12, both in grams (3.5±2.1 g, p = .015} and as a percent of total daily intake (11±1%, p = .004}. Food intake following the mating hour correlated +.95 with the anticipation ratio for wheel running, and +.27 with the ratio for motion counts. This raises the possibility that anticipation was induced by feeding rather than by copulation.

### Experiment 2

**Figure 3:**
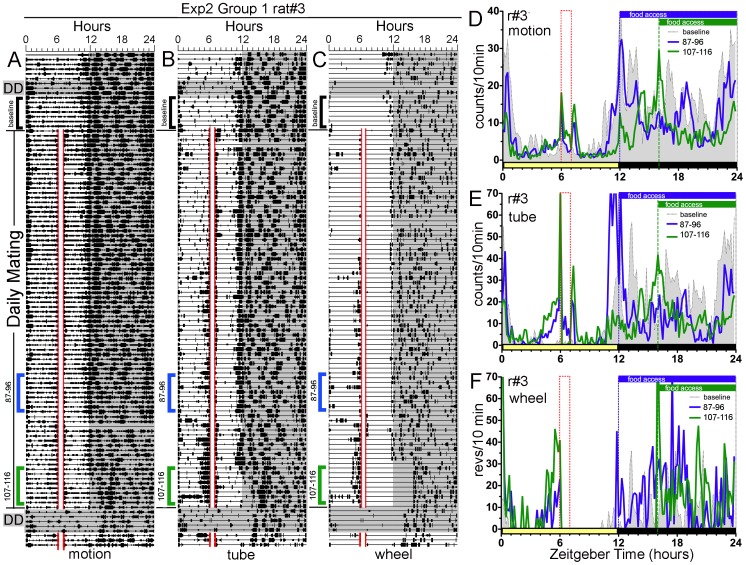
Experiment 2: One rat (Group 1, rat#3} eventually shows anticipatory activity to scheduled mating, with post-coital eating and wheel running blocked. Panels A–C. Actograms of home cage motion (A}, mating tube entry (B} and wheel running (C} in rat #3. Panels D–F. Average waveforms of activity in home cage motion (D}, mating tube entry (E} and wheel running (F} in rat#3. See Figure 2 for other plotting conventions.

Experiment 2 assessed whether daytime mating can induce anticipatory activity in male rats when post-copulatory running and eating are prevented until lights-off. In this experiment, Group 1 rats, like those in Experiment 1, showed few intromissions during the first 2 scheduled mating sessions, and a rapid increase thereafter, stabilizing at 30±6/session/rat (range 21±5 to 37±9/session across rats}. The number of ejaculations observed averaged 0.5±.1/session/rat. Despite the sustained copulatory activity, with ejaculations approximately every other session, none of the rats exhibited activity in anticipation of the mating hour, in any of the 3 activity measures, during the first 30 days of testing (e.g., Figs. 2, 3}. During the second month, activity during the 3 h preceding daily mating increased significantly in one rat (rat #3, Fig. 3}, in all 3 measures. Anticipatory activity was marginal in one other rat and entirely absent in the remaining four rats (e.g., Fig. 2a–c}. The proportion of daily activity (excluding ZT6-12} occurring in the ZT3-6 time block in rat #3 increased from 3.5%, 1.3% and 0% (for motion, tube and wheel counts, respectively} during baseline to 5.7%, 8.5% and 8%, respectively, during days 51–60 of scheduled mating (all differences significant at p<.001}. However, on the first day of DD, after the last day of scheduled mating, presumed mating anticipatory activity was absent (Fig. 3a–c}. On the first day of LD after 5 days of DD, activity reappeared in the ZT3-6 time block, in all 3 measures, but particularly strongly in the access tube measure (Fig. 3}. In the rats that did not show significantly increased activity prior to the mating hour, one rat showed some activity at the prior mating time on the first day of DD, but the other 4 rats showed only sporadic activity not significantly different from baseline activity levels expressed during the DD test conducted prior to scheduled mating.

**Figure 4:**
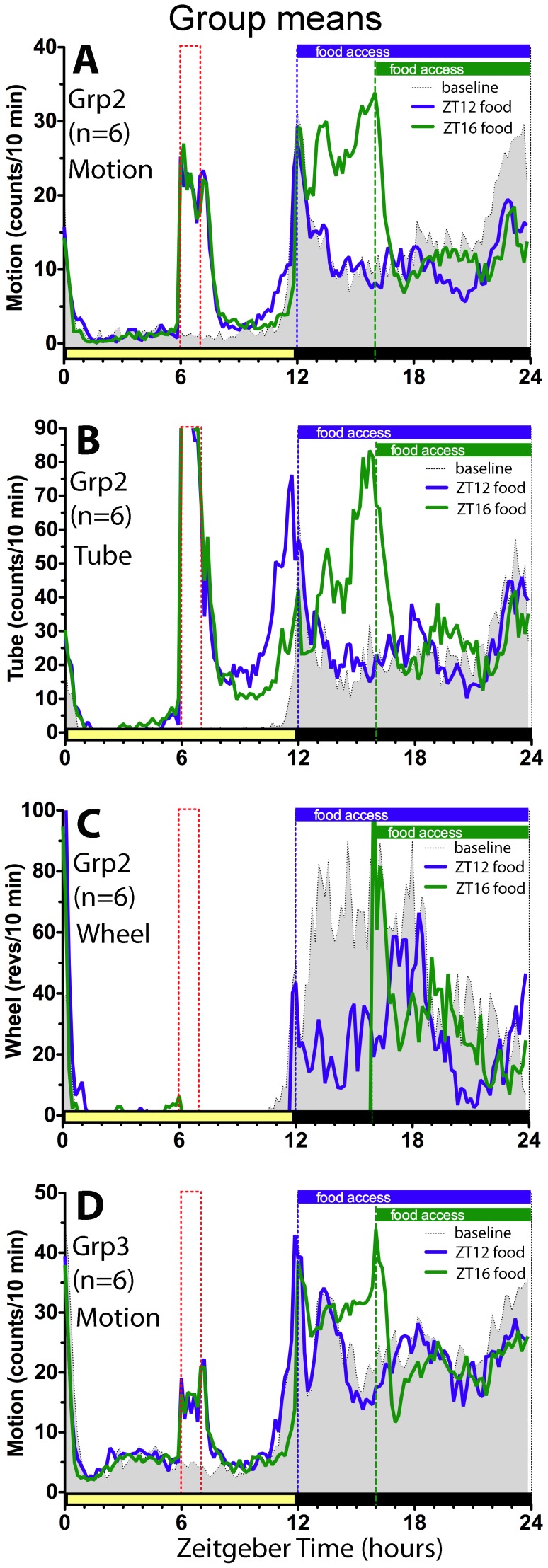
Experiment 2: Rats exposed only to sight, sound and smell of estrous females do not exhibit anticipatory activity. Group average waveforms: Panels A–C, home cage motion, mating cage access tube and running wheel activity, respectively, in Group 2 rats (n = 6}. Panel D, home cage motion in Group 3 rats (N = 6}. The shaded curves represent baseline prior to scheduled daily exposure to estrous females. The heavy (blue} curves represent scheduled exposure days 51–60. The green curves represent days 71–80. See Figure 2D–F for other plotting conventions.

Group 2 rats were exposed to the sight, sound and smell of estrous females from ZT6-7 each day, but were separated from the females by a vented gate. These rats spent the majority of the hour in the access tube interacting with the females through the gate. They exhibited clear signs of sexual arousal, including penile grooming and scent sampling by pressing their snouts against the vented gate. Despite the obvious and sustained arousal, Group 2 rats did not exhibit increased activity prior to ZT6 on any day of the experiment, in any of the 3 measures (Fig. 4a–c}.

Group 3 rats were not directly exposed to female rats, but did exhibit increased home cage activity during the scheduled mating hour (Fig. 4d}, presumably in response to olfactory and auditory stimuli associated with mating in the other cabinets. Occasional visual observations revealed behaviors similar to those observed in rats preparing to mate, including penil grooming and scent sampling by pressing their snouts against the cage tops. Despite the behavioral arousal, Group 3 rats did not show increased home cage activity prior to the mating hour.

In this experiment, running wheels in Groups 1 and 2 were locked for either 5 h or 9 h following the mating hour, and food in all 3 groups was limited to hours 1–12 or 4–12 of the dark period, to eliminate post-copulatory running or eating as potential confounds. This feeding and wheel access schedule was sufficient to induce apparent food anticipatory activity in both the motion and tube measures (Figs. 3, 4}. When food and wheels were made available *ad-libitum* in DD, all three activity measures free-ran, with onset of the daily active period delayed by about 3-h relative to both the previous time of lights-off and to the onset of activity during the 3 day DD test prior to the mating schedule (Figs. 2,3}.

### Experiment 3

#### Scheduled nighttime mating

**Figure 5:**
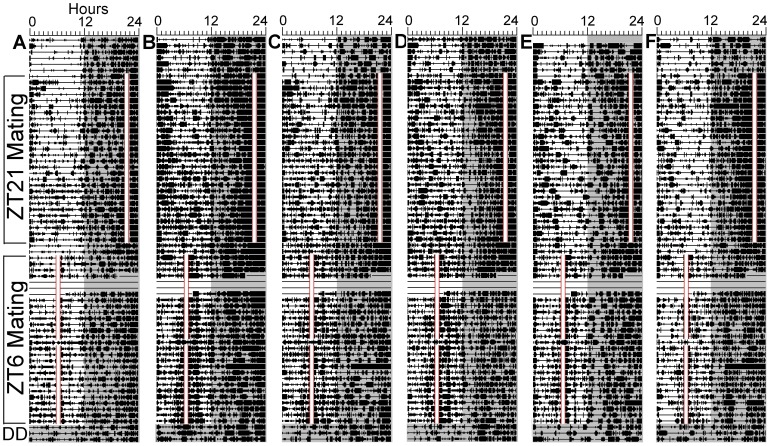
Experiment 3: Rats anticipate both late night (ZT21} and midday (ZT6} scheduled mating when provided access to a single female for a full hour to increase ejaculation rates. Each actogram represents total activity (the sum of activity counts registered by the overhead motion sensor and the two tube sensors} in 6 of 8 rats (A–F, corresponding to rats 1–6 in Figure S3}. The daily mating hour is denoted by the opaque vertical bars outlined in red. See Figure 1 for other plotting conventions.

In Experiments 1 and 2, the rats were limited to 10 minute with each of 3 females. That procedure ensured exposure to sociosexual stimuli and sustained behavioral arousal and motor activity across an entire hour, but yielded low rates of ejaculations. With post-copulatory feeding and running prevented in Experiment 2, anticipatory activity was evident in only 2 of 6 rats, and not before the second month of testing. To determine whether anticipatory rhythms might emerge more quickly and reliably if ejaculations occurred on more days, rats (N = 8} in Experiment 3 were tested at night, when ejaculation latency is significantly shorter (e.g., 34}, and were given up to 25 mins per session with a single female before switching females if no ejaculation occurred. With this procedure, ejaculations were evident on 24.5±0.6 out of 28 sessions (range  = 23–26}, with latencies averaging 11.5±1.1 mins (Fig. S1}. Anticipatory activity of individual rats is illustrated by actograms representing total activity counts (the sum of the three activity sensors; Fig. 5}, and by scatterplots (with medians and ranges} of anticipation ratios for each of the last 7 days of baseline and the last 7 days of scheduled nocturnal mating (Fig. S3}. Rank tests for these two sets of days revealed significantly increased anticipation ratios during the last week of scheduled nocturnal mating for general activity (overhead motion sensors, 7 of 8 rats} and for activity in the mating cage access tube (significant in 3 of 6 rats; 2 of 8 rats did not have activity sensors in either access tube}. All but one rat showed significant anticipatory activity in at least 1 of these 2 measures. Anticipation ratios for activity in the tube to the locked cage were significantly decreased in 1 rat and not significantly different from baseline in the others (Fig. S3}.

**Figure 6:**
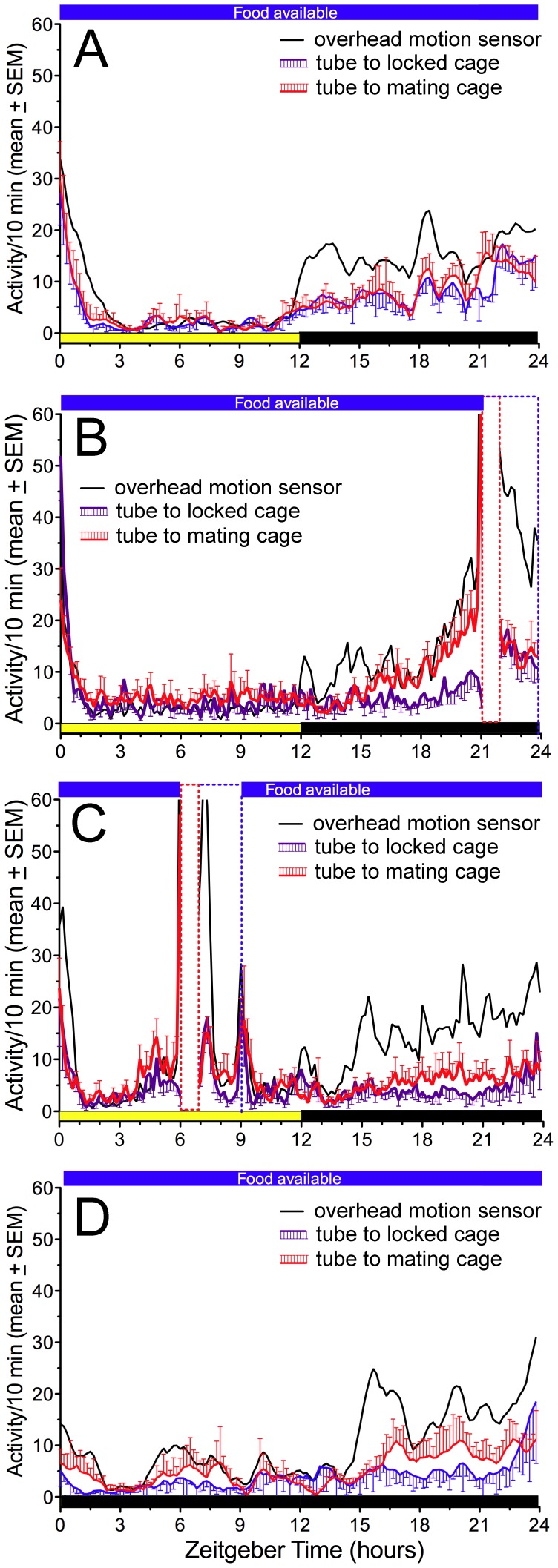
Experiment 3: Group mean (±sem} waveforms illustrating anticipatory activity in response to scheduled mating. A. Baseline week, no scheduled mating. B. Week 4, late night mating (ZT21}. C. Week 4, daytime mating (ZT6}. D. Day 2 of constant dark without scheduled mating, after the daytime mating schedule. The black curve represents activity detected by the overhead motion sensor. The purple curve (with error bars below} represents activity in the tube to the locked cage. The red curve (with error bars above} represents activity in the tube to the mating cage. Scheduled mealtime is denoted by the opaque vertical bars with dashed red borders. The time of food availability is indicated by the heavy blue line at the top of each figure.

**Figure 7:**
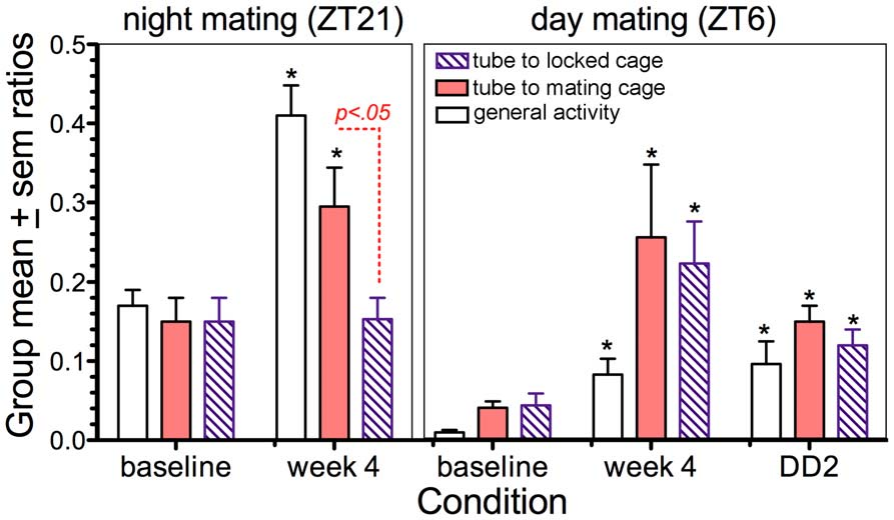
Experiment 3: Group mean (±sem} mating anticipation ratios. The ratios were calculated by dividing activity during the 3 h prior to the mating hour by total daily activity, excluding the mating hour and the 2 h immediately following. Stars denote significantly different from baseline for that activity measure (p<.05}. Ratios for activity in the tube to the mating cage were significantly different from ratios for activity in the tube to the locked cage in the ZT21 mating condition (red dashed line}, but not in the ZT6 mating condition.

Comparisons of activity in the two tubes clearly show that the anticipation counts (Fig. 6b} and ratios (Figs. 7, S3} were significantly greater for the mating cage access tube. A significant preference for this tube was not evident during the daily light period (t_(5}_ = 0.36, p = .72} or during baseline recordings prior to the mating schedule (t_(5}_ = 0.17, p = .86}. This indicates that mating restricted to a fixed time at night can induce a place preference that is specific to the mating time.

#### Scheduled daytime mating

When the mating schedule was shifted to the middle of the light period (ZT6} for 28 days, ejaculation rates decreased slightly to 20.2±1.3 out of 28 sessions (range  = 16 to 24 across rats}, and ejaculation latencies increased markedly to 21.9±2.8 minute (t_(5}_ = 4.04, p<.01 by contrast with nighttime mating; Fig. S1}. All but one of the rats exhibited significant anticipatory activity, as quantified by anticipation ratios, in each of the 3 activity measures (Figs. 7, S3}. However, the total amount of anticipatory activity was substantially lower than during nocturnal mating (Fig. 6c}. This was particularly evident in general locomotor activity. Also, the amount of anticipatory activity in the tube to the mating cage, expressed in 3 h total counts or as an anticipation ratio, did not differ significantly from activity in the tube to the locked cage (Figs. 6, 7, S3}. Thus, while the rats did anticipate mating time, they were much less active and on average displayed no consistent preference for the tube to the mating cage. During three days of DD without mating, the anticipation rhythm persisted (Figs. 5, 6d, 7}. Unlike in Experiment 1, the amount of anticipatory locomotor activity did not differ significantly on the first day of DD by contrast with the last week of LD.

#### Female behavior

The female rats were primed with estrogen and progesterone and were sexually receptive during most but not all sessions. Females that were not receptive within 25 minute s were replaced for the remainder of that session. Video recordings were used to quantify lordosis in 6 female rats, during 2 day and 2 night sessions. Lordosis was observed in all but 2 night recordings and 2 day recordings (i.e., in 83% of 24 sessions scored}. There was no evidence for a day-night difference in either the lordosis quotient (the ratio of lordosis responses to mount attempts}, or the lordosis intensity (Fig. S2}. These results are consistent with earlier reports that female receptivity does not vary with time of day in ovariectomized, steroid-primed female rats [40]. Day-night differences in male ejaculation latency and anticipatory activity thus do not appear to be caused by differences in female receptivity.

## Discussion

This study demonstrates that scheduled mating can induce daily rhythms of anticipatory behavior in male rats and identifies food as a potential confound variable, time of day as a modulating variable, and ejaculations as a mediating variable. The results support a hypothesis that circadian oscillators driving anticipatory rhythms can be entrained by activation of reward circuits, and that significant metabolic perturbations such as those associated with deprivation schedules or high caloric snacks are not required.

Experiments 1 and 2 were methodologically matched with the exception of the availability of food immediately following the 1-h mid-day (ZT6} mating session. In Experiment 1, all 6 male rats exhibited clear anticipation of the daily mating session by the second week of testing. After mating sessions, the rats exhibited a burst of running when returned to the home cage, and significantly elevated food intake over the next 5 h. The amount of anticipatory activity was significantly correlated with the amount eaten during this 5 h. In Experiment 2, wheel running and food intake were prevented until lights-off, and none of 6 rats exhibited anticipatory activity during the first month of testing in any of the three activity measures. Consistent anticipatory activity emerged in one rat during the second month of testing, but only one other rat showed anticipation, and only sporadically, by day 80 of testing. These results indicate that eating, presumably stimulated by arousal and activity during the 60 min daily mating session, either caused or contributed to the induction of anticipatory activity observed in Experiment 1. The burst of wheel running on return to the home cage might have also contributed, although previous studies have shown that scheduled running does not induce anticipatory activity in rats [42]. All of the rats in Experiments 1 and 2 were behaviorally alert during the 60 min tests, including those rats that could only see, hear and smell the estrous females, thus it is clear that behavioral arousal alone is not sufficient to induce anticipatory rhythms, even when the arousal stimulus has strong incentive properties. Similar results have been reported for male mice provided access to a female mouse once daily late in the light period [36].

The fact that one rat in Experiment 2 did eventually show consistent anticipatory activity to the daytime mating sessions, despite being prevented from eating until lights-off, is suggestive that circadian oscillators driving anticipatory rhythms can be entrained by sexual reward. However, this result is considered equivocal because the anticipatory rhythm in this rat was absent during 3 days in constant dark without scheduled mating, and then reappeared at the usual time when LD was reinstated. Circadian clock control of anticipatory rhythms is inferred from the persistence of these rhythms in the absence of potential external time cues. Anticipatory activity in this rat therefore failed the first criterion for circadian clock control, and instead appeared to depend on light-onset as a timing cue, a characteristic of interval timing [5], [43]. There is an extensive literature on daily food anticipatory rhythms in rats, and a similar dependence of anticipation on LD cues has never been observed, to our knowledge.

Experiments 1 and 2 were designed to keep the male rats actively engaged in copulatory behavior for an entire hour. This was accomplished by providing each rat with access to three different estrous female rats during each 60 min session, with a 10 min rest between females. All of the rats exhibited mounting and intromissions in each session, but ejaculations were observed on less than half of the days, presumably due to the 10 min limit with each female. Although mounting and intromissions have been shown to be rewarding, ejaculations are by various measures more rewarding [44]–[48]. Therefore, it is possible that the mating schedule used in Experiments 1 and 2 was not sufficiently rewarding to induce anticipatory rhythms (i.e., the reward ‘dose’ was insufficient}. There is also evidence for a daily rhythm of sexual reward, with a circadian maximum at night and a circadian minimum in the second half the light period [37]. The circadian minimum defined in that study is close to the midday hour (ZT6} that we chose for scheduled mating, to be consistent with studies of daily food anticipatory rhythms (e.g., [3]).

Experiment 3 was therefore designed to assess reward ‘dose’ and time of day effects. To maximize the ‘dose’, the rats were provided with access to one estrous female for up to an hour so that ejaculations were achieved in most if not all sessions. No more than 1 ejaculation per day was permitted, to minimize the chance that sexual satiety would inhibit mating behavior on the next day. This procedure was successful at both the late-night and mid-day mating times, and significant anticipatory activity was evident in all but one rat to both the night and the day sessions. The rhythm of anticipatory activity at both times persisted during constant conditions (no light or mating session}, thereby meeting criterion for circadian clock control. This result demonstrates that copulatory behavior, specifically ejaculation, is a sufficient stimulus for inducing circadian rhythms of anticipatory behavior, and supports a hypothesis that reward circuits participate in the timing mechanism for anticipatory rhythms, either as a site of circadian oscillators, or as a source of entrainment signals for oscillators elsewhere.

Although the rats anticipated mate access during both the nighttime and the daytime mating schedules, there was a marked difference in the amount of anticipatory activity and in the apparent preference of the rats for the mating access tube during the hours immediately preceding mate access. Total anticipatory activity was substantially greater prior to the nighttime mating sessions. This is likely due in part to inhibitory effects of light on activity in nocturnal rats, and suppression of activity during the normal sleep phase by the LD-entrained SCN circadian pacemaker. Daily variations in the magnitude of perceived reward may also contribute. This is suggested by the observation that anticipatory activity was strongly biased toward the mating access tube during the nighttime schedule but not the daytime schedule. This bias is reminiscent of a conditioned place preference (CPP}, which can be induced in male rats by pairing ejaculations with one of two neutral chambers (e.g., [37]). CPPs have been used to operationally define reward [49]. Rats exhibit a robust CPP to mating early in the night, but no significant CPP to mating in the mid-to-late day, suggesting a circadian rhythm of sexual reward, with maximal reward at the time of day when male rats are most likely to encounter an estrous female [24], [37]. The lower level of anticipation and lack of bias toward the mating access tube exhibited by our rats during daytime mate access may represent further evidence for a daily rhythm of sexual reward in this species. Presumably mating in the day does activate reward circuits (otherwise mating attempts would extinguish}, but the magnitude of sexual reward at ZT6 may be below the level of detection by a CPP behavioral assay.

By contrast with anticipation of a daily mating opportunity, the magnitude and duration of anticipation of a daily meal does not show the same degree of variation with time of day, and is equally robust to meals scheduled in the day or the night (e.g., [50]). This is likely because caloric restriction stimulates activity, and also potentiates the apparent reward value of natural and drug rewards [51], [52]. It is also possible that food and sexual reward are differentially modulated by circadian factors [24], [37].

Daytime restricted feeding schedules induce anticipatory activity rhythms in rats without shifting the SCN pacemaker [53]–[55], and these rhythms persist robustly in SCN-ablated rats, indicating control by non-SCN oscillators [6], [7]. We observed apparent compression of nocturnal activity in Experiment 2 when food was limited to hours 3–12 of lights off, but there was no evidence for shifting of light-entrained rhythms by the mating schedules (nocturnal activity-onset was delayed, but the end of activity was not}. Thus it is likely that sex anticipation is also mediated by non-SCN oscillators, although this remains to be confirmed in rats or mice lacking a functional SCN. Our results do invite speculation that oscillators controlling mating anticipatory activity are separate from oscillators responsible for the daily rhythm of sexual reward. This is suggested by the apparent dissociation between the rhythm of anticipation and the rhythm of reward, inferred from the anticipation and place preference data in Experiment 3. In that experiment, four weeks of mid-day mating induced anticipatory activity but did not reset the daily rhythm of ejaculation latency or reestablish the preference for the mating cage access tube that was evident during scheduled nocturnal mating.

Daily rhythms of circadian clock gene expression are evident in limbic reward circuits, and these can be shifted by restricted feeding schedules or dopaminergic stimuli, neither of which reset the SCN pacemaker in the presence of a daily LD cycle [18], [23], [25], [27]. It will be of interest to determine whether daily mating schedules also reset mesolimbic circadian oscillators. One possibility is that mesolimbic oscillators contribute to food and mating anticipatory rhythms, while the SCN pacemaker controls the daily rhythm of sexual reward, perhaps by modulating the response of mesolimbic reward neurons to sensorimotor correlates of ejaculation. Food anticipatory rhythms persist following complete ablation of the nucleus accumbens [56], a core structure in the reward system, but whether such lesions affect anticipation of mating is unknown, and a necessary role for other components of the reward system in anticipatory rhythms has yet to be examined.

In conclusion, the results of this study show that successful mating activity can function as a nonphotic, social stimulus capable of adjusting the circadian behavioral program by inducing daily peaks of anticipatory activity, qualitatively if not quantitatively similar to those induced by restricted daily feeding schedules. There has been renewed interest in social stimuli as circadian zeitgebers [57]–[59], and the present results suggest that social synchrony can involve circadian oscillators outside of the light-entrainable SCN pacemaker. Comparative studies of scheduled mating and restricted feeding should reveal whether natural rewards affect a common circadian oscillator for anticipatory appetitive behaviors, or whether different populations of circadian clock cells are recruited by different reward stimuli.

## Supporting Information

Figure S1 **Group mean (±sem} latencies to intromission (IM, solid curves} and ejaculation (EJ, dashed curves}, during scheduled midday (ZT6, blue curves} and late night (ZT21, red curves} mating in Experiment 3.**
(TIF)

Figure S2 **Group mean (±sem} lordosis quotients and lordosis intensity scores for late night (ZT21} and daytime (ZT6} mating sessions in Experiment 3.**
(TIF)

Figure S3 **Scatterplots of mating anticipation ratios for individual rats (Rat1-Rat6} during the last 7 days of baseline and the last 7 days (week 4} of restricted mating access in Experiment 3.** The left column of panels represents ratios for overhead motion sensor data. The middle column represents ratios for activity in the tube to the mating cage. The right column represents ratios for activity in the tube to the locked cage that contained litter from the mating cage. Two additional rats with motion sensor data but no tube sensor data are not shown. Within each panel, grey shading denotes late night (ZT21} mating and yellow shading denotes daytime (ZT6} mating. Median values for each 7-day set of ratios are indicated by horizontal bars. Statistical significance (Mann-Whitney U tests} of differences in ratios between baseline and week 4 for each mating time and each variable is denoted by one (p<.05} or two (p<.01} stars.
(TIF)
