# Supplementary figures and images for: Scheduled Daily Mating Induces Circadian Anticipatory Activity Rhythms in the Male Rat
Source: PLoS One. 2012 Jul 25;7(7):e40895. doi: 10.1371/journal.pone.0040895 (PMC3405034; doi:10.1371/journal.pone.0040895)

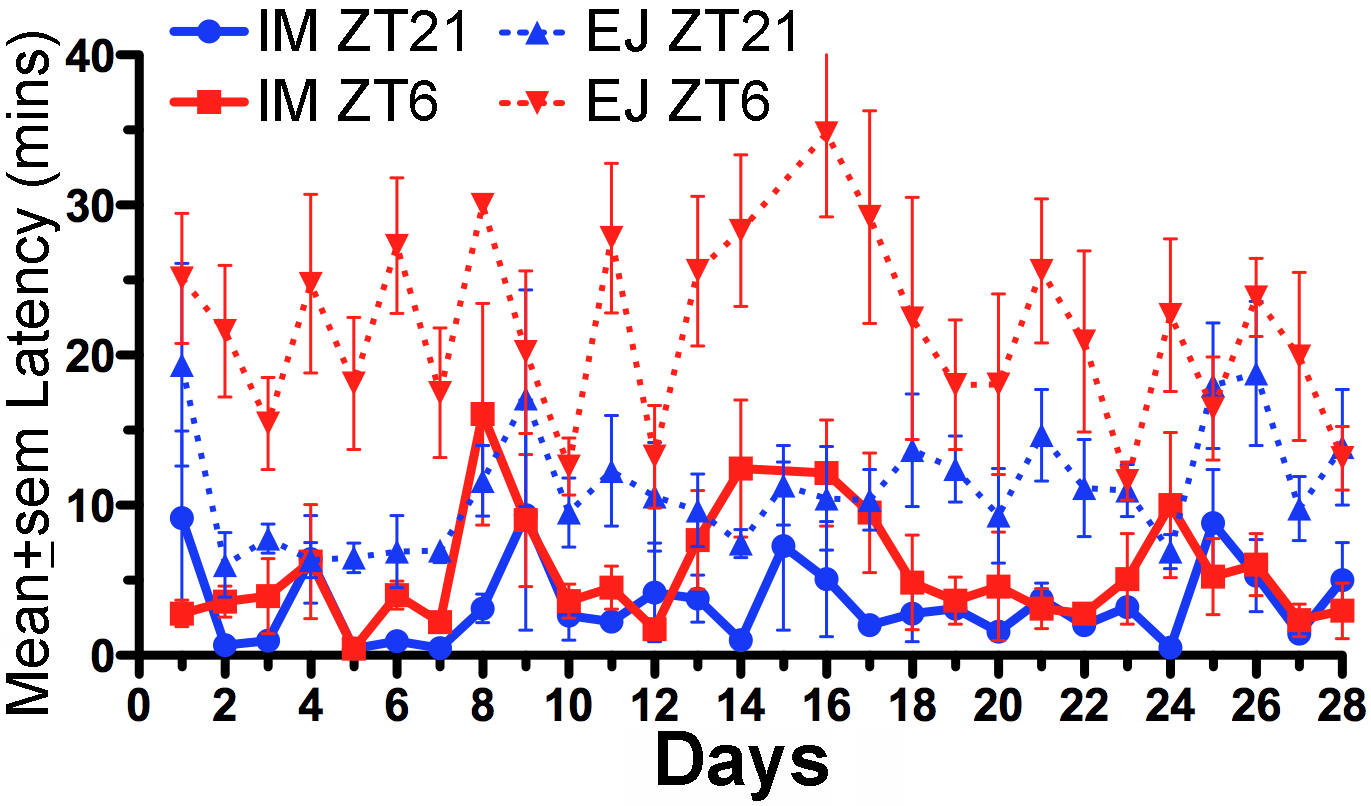

Supplement: Figure S1 — Group mean (±sem} latencies to intromission (IM, solid curves} and ejaculation (EJ, dashed curves}, during scheduled midday (ZT6, blue curves} and late night (ZT21, red curves} mating in Experiment 3. (TIF) [file pone.0040895.s001.tif]

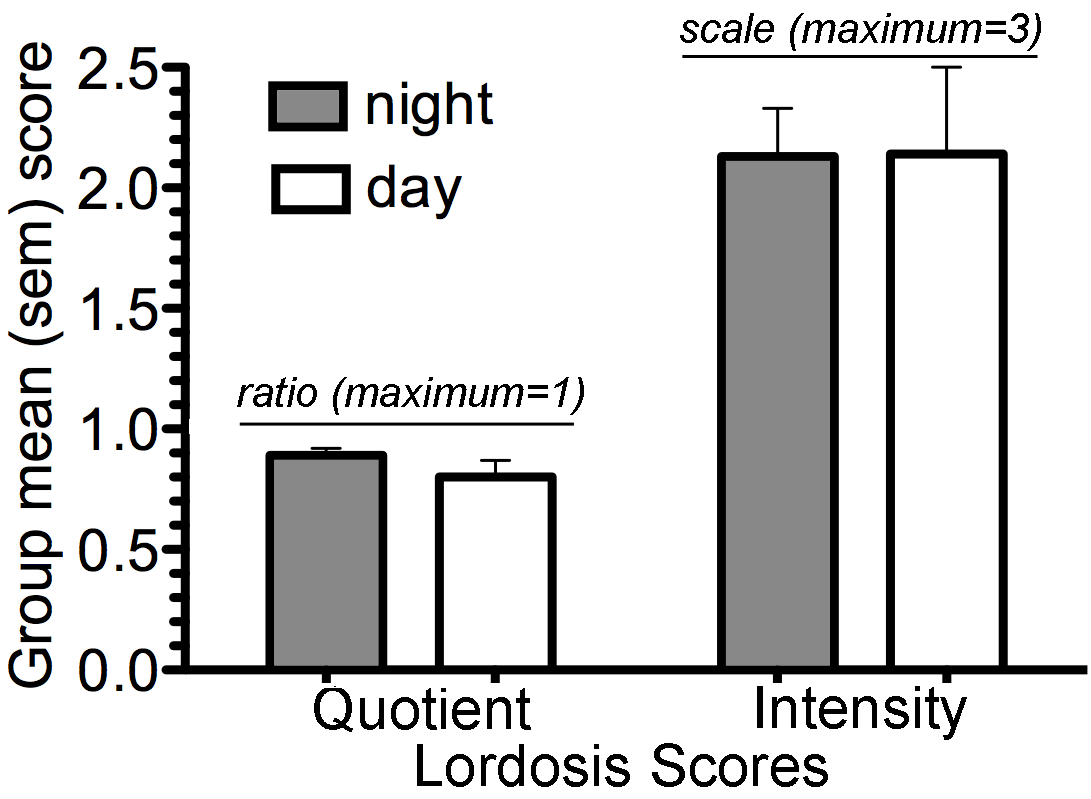

Supplement: Figure S2 — Group mean (±sem} lordosis quotients and lordosis intensity scores for late night (ZT21} and daytime (ZT6} mating sessions in Experiment 3. (TIF) [file pone.0040895.s002.tif]

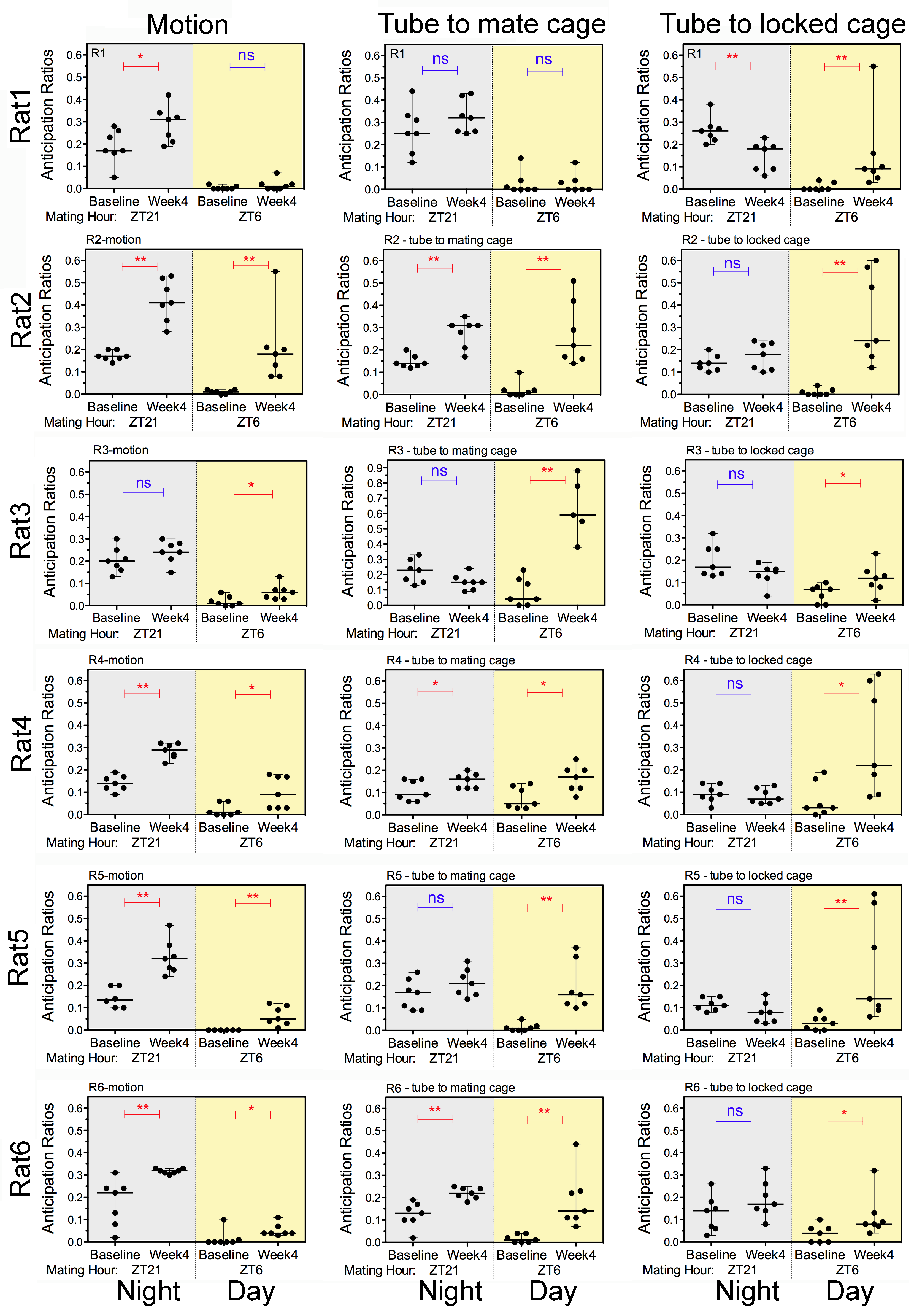

Supplement: Figure S3 — Scatterplots of mating anticipation ratios for individual rats (Rat1-Rat6} during the last 7 days of baseline and the last 7 days (week 4} of restricted mating access in Experiment 3. The left column of panels represents ratios for overhead motion sensor data. The middle column represents ratios for activity in the tube to the mating cage. The right column represents ratios for activity in the tube to the locked cage that contained litter from the mating cage. Two additional rats with motion sensor data but no tube sensor data are not shown. Within each panel, grey shading denotes late night (ZT21} mating and yellow shading denotes daytime (ZT6} mating. Median values for each 7-day set of ratios are indicated by horizontal bars. Statistical significance (Mann-Whitney U tests} of differences in ratios between baseline and week 4 for each mating time and each variable is denoted by one (p<.05} or two (p<.01} stars. (TIF) [file pone.0040895.s003.tif]
